# Corticosteroid treatment for acute/acute-on-chronic experimental and naturally occurring pancreatitis in several species: a scoping review to inform possible use in dogs

**DOI:** 10.1186/s13028-021-00592-0

**Published:** 2021-07-13

**Authors:** Kari-Anne Bjørnkjær-Nielsen, Charlotte Reinhard Bjørnvad

**Affiliations:** 1 Dyrlægehuset Skanderborg, Ladegaardsbakken 16, 8660 Skanderborg, Denmark; 2 Department of Veterinary Clinical Sciences, Faculty of Health and Medical Sciences, University of Copenhagen, Dyrlægevej 16, 1870 Frederiksberg C, Denmark

**Keywords:** Canine, Dexamethasone, Glucocorticoid, Hydrocortisone, Steroid

## Abstract

Acute pancreatitis in dogs is a prevalent disease characterised by mild to severe inflammation. Treatment with anti-inflammatory corticosteroids has been widely debated but is not generally recommended in veterinary medicine. The objective of the present study was to present current evidence on the effect of corticosteroid treatment for acute/acute-on-chronic pancreatitis across species. These findings were then used to evaluate if and how corticosteroid treatment could influence disease outcome in canine acute/acute-on-chronic pancreatitis. A scoping review was performed by searching the Agricola, CAB Abstracts, MEDLINE and Embase databases to identify relevant articles published before June 24, 2021. The inclusion criteria were English language, original research published in a peer-reviewed journal, and investigation of corticosteroid treatment effects on acute/acute-on-chronic pancreatitis by the outcome parameters clinical score, circulating CRP level, hospitalisation duration, mortality and pancreas histopathology. Research on any species was considered. Studies were rated based on the level of evidence, and methodological quality was evaluated based on similarity between groups at baseline, risk of bias and study group size. The reporting method was based on the PRISMA extension for scoping reviews. One thousand nine hundred fifty-four studies were identified, and 31 met the inclusion criteria. Five were canine studies, with 4 investigating experimentally induced pancreatitis; 5 were human clinical studies; and 21 were rodent studies of experimentally induced pancreatitis. The level of evidence ranged between randomised controlled trials and case series, the estimated risk of bias ranged from low to high, and the sample sizes ranged from very small to moderate. Evidence indicates that adding corticosteroid to symptomatic treatment of acute/acute-on-chronic canine pancreatitis could have a positive influence on disease outcome. However, the analysed evidence was based on several species, including both naturally occurring and experimentally induced pancreatitis; thus, the authors suggest that large randomised controlled studies should be performed in dogs with spontaneously occurring acute/acute-on-chronic pancreatitis to further elucidate a potential benefit of corticosteroid treatment.

**Supplementary Information:**

The online version contains supplementary material available at 10.1186/s13028-021-00592-0.

## Background

Canine acute pancreatitis is a common disease in veterinary practice, and the clinical presentation can vary from subclinical or mild nonspecific clinical signs to severe life-threatening disease [1].

Our understanding of the pathophysiology of acute pancreatitis is largely extrapolated from human clinical studies and experimental animal models. An apical block is believed to cause intracellular fusion of zymogen granules and lysosomes, which leads to the activation of trypsinogen and release of cathepsin-B and other pancreatic enzymes within acinar cells. These changes activate the apoptotic cascade and cause inflammation, which is manifested by neutrophil migration to the pancreas as well as probable complement activation and a “cytokine storm” that further contributes to inflammation [2, 3].

The fact that pancreatitis is an inflammatory condition is supported by increases in parameters associated with inflammation, such as C-reactive protein (CRP), Interleukin 6 (IL-6) and Tumor necrosis factor α (TNF-α) [4], as well as histologic findings [5]. Although universally standardised criteria are not available for the histologic classification of pancreatitis in dogs [6, 7], canine acute pancreatitis has been defined by Watson et al. as pancreatic necrosis that frequently presents with a neutrophilic infiltrate but without fibrosis or chronic inflammation [8]. This definition is consistent with Newman et al. [9] who suggest that neutrophilic inflammation is often associated with pancreatic necrosis and/or peripancreatic fat necrosis. These findings are considered potentially completely reversible. The presence of permanent histopathological changes, such as fibrosis and acinar atrophy, suggest chronic pancreatitis, and a mononuclear or mixed inflammatory infiltrate is expected [8–10].

In the clinical setting, distinguishing between acute and chronic canine pancreatitis is difficult. Truly acute disease cannot be distinguished from acute-on-chronic pancreatitis [11], and histopathological assessments are infrequent due to the cost and/or possible associated morbidity [7, 12, 13]. Furthermore, studies show that in many histopathological samples, acute and chronic changes are found concurrently [10, 14], which make the distinction between acute and chronic pancreatitis less relevant. This mix of acute and chronic disease has led authors to conclude that chronic pancreatitis may result from recurrent acute disease [7] and that some apparently acute pancreatitis cases are in fact acute exacerbations of previously unrecognised chronic disease [11].

Therefore, this study includes cases of acute-on-chronic as well as truly acute canine pancreatitis, which is referred to as CAP, when discussing the condition in dogs with acute symptoms of pancreatitis.

Canine acute pancreatitis can result in acute life-threatening systemic complications, such as disseminated intravascular coagulation and multiple organ failure [2]. Ongoing inflammation can result in progressive fibrosis and loss of exocrine and/or endocrine tissue, thus causing the development of exocrine pancreatic insufficiency and/or diabetes mellitus [2, 11]. Hence, initiating the optimal treatment for CAP as soon as possible can have considerable positive results.

Although multiple risk factors and rare underlying causes have been identified [1, 7], most cases of CAP are considered idiopathic and do not present an underlying disease for treatment [6]. The mainstay treatment recommended for CAP includes aggressive fluid therapy, a low-fat diet, antiemetics and analgesic agents, including opioids [15, 16].

As a confirmed inflammatory disease, it is noteworthy that anti-inflammatory medications are rarely discussed for the treatment of CAP. A search of the literature shows that although nonsteroidal anti-inflammatory agents (NSAIDs) are generally not recommended [15–20], a consensus has not been reached regarding the use of corticosteroids. Corticosteroids are generally contraindicated because of the risk of gastric ulcerations and reduced reticuloendothelial activity [19]. Recent human case studies have indicated that corticosteroids may even induce acute pancreatitis [21, 22]. In dogs with hyperadrenocorticism, one study reported increases in canine pancreatic-specific lipase (Spec-cPL) concentrations without any obvious clinical evidence of pancreatitis [23]. However, dogs in this study were not evaluated for evidence of subclinical pancreatitis. In another study, hyperadrenocorticism resulted in an increased prevalence of ultrasonographic pancreatic hyperechogenicity compared with normal dogs, despite that they had normal Spec cPL concentrations and no obvious signs of clinical pancreatitis [24]. It should also be noted that hyperadrenocorticism has been identified as a risk factor for developing fatal acute pancreatitis [25]. However, a study in 2018 on healthy beagles showed that even immunosuppressive doses of prednisolone did not induce clinical or histological evidence of pancreatitis [26]. Several authors have indicated the lack of evidence suggesting that steroids reduce pancreatic inflammation [19], and some have narrowed this statement to apply to dogs only [20]. In contrast, a recent publication suggested that morbidity may be improved and/or mortality may be reduced in CAP by using low-dose corticosteroids [16].

This study performed a scoping review of peer-reviewed literature to present current evidence on the effects of corticosteroid treatment on acute/acute-on-chronic pancreatitis across species to provide recommendations regarding the use of corticosteroids for CAP.

## Search strategy

### Methods

The method of reporting was performed according to the Preferred Reporting Items for Systematic Reviews and Meta-Analyses (PRISMA) extension for scoping reviews [27]. A review protocol including inclusion and exclusion criteria for the screening processes as well as a search strategy was defined prior to starting this review; this protocol was dated May 12, 2019 and is available on request. The clinical research question, which was formulated by the PICO method [28], was as follows: In dogs with acute/acute-on-chronic pancreatitis receiving standard treatment, could adjuvant therapy with corticosteroids improve the disease outcome relative to that of standard treatment alone? Markers of improved disease outcome were clinical score (as defined by the authors), circulating CRP levels, hospitalisation duration, mortality rate and pancreas histopathology.

### Eligibility criteria

#### Types of studies

Studies reporting how corticosteroid treatment affects acute/acute-on-chronic pancreatitis with regard to at least one of the abovementioned markers of disease outcome were considered. As a sparse amount of literature was expected, studies were not excluded based on the study design or restricted by publication date; however, the language was restricted to English. Articles for which full text was not available or that were not peer-reviewed according to the publisher’s webpage or the article’s cover page were excluded. Conference abstracts were excluded since they are frequently publicly unavailable. Reviews and meta-analyses were excluded because the relevant original studies were included when relevant and available instead.

#### Types of participants

As few studies in dogs were expected, exclusions based on species were not performed. Individuals suffering from acute/acute-on-chronic pancreatitis that spontaneously occurred or was experimentally induced were included. In vitro studies were excluded. Human studies on autoimmune pancreatitis were excluded because this disease has not been documented to cause acute pancreatitis in dogs [7]. Human paediatric studies were excluded because a strong genetic background is suspected [29], which is unknown in dogs. Human studies in which acute pancreatitis was found to be a sequela to or part of another specific disease (e.g., systemic lupus erythaematosus) or treatment thereof (medical or surgical) were excluded. Concomitant biliary disease and alcoholism in humans were accepted because they are considered predisposing factors.

#### Types of intervention

Studies reporting corticosteroid treatment as a single treatment or an adjuvant to standard treatment of acute/acute-on-chronic pancreatitis were considered. No exclusion due to corticosteroid type or dose was conducted. However, studies involving anabolic steroids as well as endogenous corticosteroids were excluded. Studies reporting the preventive effect of corticosteroids given prior to or simultaneously with inducing experimental acute pancreatitis and studies reporting corticosteroid treatment as a cause of pancreatitis were excluded.

#### Types of comparison/control

Individuals with acute/acute-on-chronic pancreatitis following the same supportive treatment protocol apart from corticosteroid treatment were used as model controls.

#### Types of outcome

The primary outcome was the corticosteroid treatment effect based on the clinical score (as defined by the authors), circulating CRP levels, hospitalisation duration, mortality and pancreas histopathologic evaluation.

### Information sources

A literature search was conducted by the first author (KBN) from August 21 to August 28, 2019, using the following databases: Agricola (1970–present), CAB Abstracts (1910–present), MEDLINE (1946–present), and Embase (1974–present). No limits were applied to publication year.

### Search

The following search terms were queried in the electronic databases: Steroid*; corticosteroid*; cortiso*; glucocorticoid*; dexamethason*; hydrocortiso*; predniso*; methylpredniso*; acute pancreatiti*; acute on chronic pancreatiti*; acute-on-chronic pancreatiti*. For the complete search strategy, see Additional file 1.

### Study selection

**Fig. 1:**
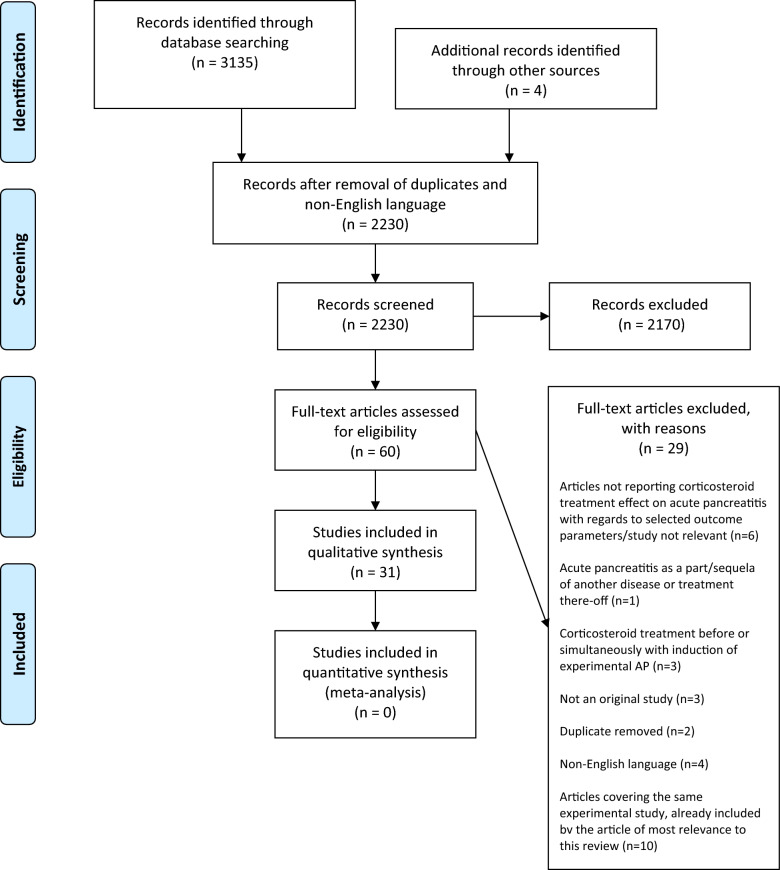
PRISMA 2009 flow diagram elucidating the inclusion and exclusion process for studies found in the database search. From Moher et al. [89]

The eligibility assessment was based on titles and abstracts in an unblinded standardised manner. Studies fulfilling the inclusion criteria and those in which criteria fulfilment could not be determined by title or abstract were retrieved as full texts and assessed by the same criteria. References of included articles as well as publications reviewing corticosteroid treatment for CAP/acute/acute-on-chronic pancreatitis from 2010 onward were screened manually for additional articles of relevance. Duplicates were manually removed. A Prisma 2009 flow diagram was used to elucidate the study selection process (Fig. 1).

### Data collection process

A data charting form (Table 1) was developed using Microsoft Word 2010 software to extract relevant information from the included studies regarding the study protocol and the results of the corticosteroid treatment effect. Ten randomly chosen studies were used to test the form and adjust the design.

**Table 1:** Summary of studies evaluating the effect of glucocorticosteroids for acute pancreatitis on clinical score, duration of hospitalization, C-reactive protein, and histopathologic score

Study	Level of evidence, study design and methodological quality (for details see Table 2)	Species	Number of subjects in groups	Acute pancreatitis type	Treatment: Type of GC, dose, duration, administration route and timing	Other treatment (GC group + model controls)	Data collection and timing, post induction unless otherwise stated	Outcome: Clinical score	Outcome: CRP	Outcome: Duration of hospitalisation	Outcome: Mortality	Outcome: Pancreas histopathology
Okanishi et al. [57]	LOE III Non-RCT Moderate estimated risk of bias, Moderate number of well characterised dogs in each group	Dogs	Model controls: 20 GC group: 45	Spontaneous AP	Prednisolone 1 mg/kg/day SC, from diagnosis to discharge	IV fluids, maropitant, famotidine, enrofloxacin, fentanyl, multivitamin solution	Daily clinical score evaluation in hospital, CRP, mortality. Follow up 1 month after diagnosis	Days until clinical score ≤ 2/3 was significantly lower in GC group (P < 0.001) (median 4 vs 7 days)	Days until CRP had reached < 2 mg/dL was significantly lower in GC group (P < 0.001) (median 4 vs 8 days)	Significantly shorter in GC group (P = 0.002) (median: 5 vs 8 days)	1-month survival was significantly higher in GC group (P = 0.005) (88.7% vs 57.9%)	NA
Studley and Schenk 1982 [39] Experiment 1	LOE II RCT, low estimated risk of bias, very small number of poorly characterised dogs in each group	Dogs	Model controls: 8 Group 1: 6 Group 2: 6	Induced AP: 10 mL bile injected into the pancreatic duct	Hydrocortisone 2.86 mg/kg IV, then 1.43 mg/kg q8h Controls: No GC Group 1: GC 6 h post induction Group 2: GC 12 h post induction	IV fluids: 5% dextrose in 0.2% saline 10 mL/kg SC q8h	Survival up to 72 h	NA	NA	NA	Survival time increased in treatment groups controls: 18.8 h Group 1: 40.2 h (P < 0.05) Group 2: 48.3 h (P < 0.03) No difference between treatment groups	NA
Studley and Schenk [39] Experiment 2	LOE II RCT, low estimated risk of bias, very small number of poorly characterised dogs in each group	Dogs	Model controls: 6 Group 1: 5 Group 2: 6	Induced AP: 10 mL bile injected into the pancreatic duct	Hydrocortisone Controls: No GC Group 1:2.86 mg/kg IV, 6 h post induction, then 1.43 mg/kg q8h Group 2: Hydrocortisone 14.3 mg/kg 6 h post induction, then q8h	Fluid replacement: 5% dextrose in 0.2% saline 10 mL/kg SC q8h	Survival up to 72 h	NA	NA	NA	Survival time increased in treatment groups Controls: 27 h Group 1: 45.6 h (P < 0.05) Group 2: 41.6 h (P < 0.05) No difference between treatment groups	NA
Attix et al. [58]	LOE III Controlled trial, high estimated risk of bias, very small number of poorly characterised dogs in each group	Dogs	Model controls: 4 GC group: 4	Induced AP: Oleic acid 5 mL/kg injected into the main pancreatic duct	Dexamethasone 0.06 mg/kg IV q24h for 8 days, start 24 h post induction	IV fluids incl. glucose for 5 days postop. Procaine penicillin G 20.000 U/kg IM q12h, Kanamycin sulfate 5 mg/kg IM q8h, NPO for 8 days	Clinical evaluation 3 times daily, histopathology at day 8, technique and results not shown, only photos	No clinical difference	NA	NA	NA	No results/conclusions for difference in histopathology among groups reported
Stewart et al. [59]	LOE III Controlled trial, high estimated risk of bias, small number of poorly characterised dogs in each group	Dogs	Model controls: 16 GC group: 25	Induced AP: A solution of trypsin and 4% sodium taurocholate injected into the main pancreatic duct	Cortisone acetate 100 mg IM, as soon as the abdomen could be closed, then 50 mg q12h for 4 days, then gradually withdrawn	None stated	Survival, pancreas histopathology when survival was assured (day 5–60), technique and results not shown	NA	NA	NA	Survival: control 6%, GC group 48%	GC group had decreased oedema and inflammation in the first few days, less apparent in animals autopsied after ≤ 5 days
Imahori et al. [60] Study 1	LOE III Controlled trial, low estimated risk of bias, small number of poorly characterised dogs in each group	Dogs	Model controls: 14 GC group: 14	Induced AP: Autologous bile injected into the pancreatic duct	Hydrocortisone 2.86 mg/kg IV, 6 h post induction, then 1.34 mg/kg q8h	Fluid treatment 5% dextrose in 0.2% saline 10 mL/kg SC q8h	72 h survival, histopathology at death or 72 h, score 0–3 (oedema, PMN leucocyte infiltration, vascular thrombosis, necrosis, haemorrhage)	NA	NA	NA	Significantly increased duration of survival in GC group (P < 0.01)	Acinar necrosis significantly decreased in GC group (P < 0.05)
Imahori et al. [60] Study 2	LOE III Controlled trial, high estimated risk of bias, very small number of poorly characterized dogs	Dogs	Model controls: 6 GC group: 5	Induced AP: Autologous bile injected into the pancreatic duct	Hydrocortisone 14.3 mg/kg at 6, 14 and 22 h post induction	Fluid treatment 5% dextrose in 0.2% saline 10 mL/kg SC q8h	Ultrastructural changes to the pancreas at 24 h	NA	NA	NA	NA	Pancreatic structural integrin better preserved in the GC group
Kaplan et al. [65]	LOE III Retrospective case control study, high estimated risk of bias, small number of fairly well characterised patients in each group	Humans	Model controls: 48 GC group: 15 GC	Spontaneous Acute oedematous: 51 Acute haemorrhagic/necrotising: 12	Hydrocortisone 100-200 mg IV every 6-8 h for 2–3 days, then tapered	Nasogastric suction, anticholinergics, antibiotics, IV fluids	Clinical evaluation, mortality	Dramatic response reported	NA	NA	Oedematous AP: all survived Haemorrhagic AP: Overall mortality 8/12 (67%) GC group 5/9 (55%)	NA
Liu et al. [40]	LOE II Randomised controlled trial, moderate estimated risk of bias, moderate number of poorly characterised patients in each group	Humans	Model controls: 53 GC group: 26	Spontaneous AP	Dexamethasone 20–30 mg then 30-60 mg/day, tapered over 3–5 days, Dextran 500 mL/day and Salvia miltiorrhiza 20–30 mL, treatment initiated at diagnosis	NPO, pancreatin secretion inhibition, antibiotics, fluids, parenteral nutrition	Mortality	NA	NA	NA	Reduced in GC group 11.54% vs 32.08% (P < 0.05)	NA
Eklund et al. [37]	LOE III Retrospective case control-study, low estimated risk of bias, small number of well characterised patients in each group	Humans	Model controls: 11 GC group: 10	Spontaneous AP	Hydrocortisone 3–400 mg/day, started 4-11d after symptom onset	Ventilation, IV fluids antibiotics (cefuroxime mostly)	CRP up to 48 h, 30-day mortality	NA	CRP significantly lower in GC group at 48 h (P = 0.043), not at 24 h	NA	30-day mortality GC group 30%, controls 36%	NA
Wang et al. [66]	LOE IV Case-series, high estimated risk of bias, moderate number of fairly well characterised patients in the group	Humans	Model controls: 0 (literature reference) GC group: 32	Spontaneous AP	Dexamethasone 0.5–1 mg/kg q24h for3-5 days, starting 8 h to 4 days after onset of symptoms Dextran-40 500–1000 mL/day for 7 days	Nasogastric tube decompression, oxygen, IV fluids, antibiotics (imipenem)	Clinical evaluation, mortality	4-8 h after treatment start pain was relieved	NA	NA	GC group: 12.5% literature: 40%	NA
Wan et al. [38]	LOE II RCT, low estimated risk of bias, moderate number of well characterised patients in each group	Humans	Model controls: 35 GC group: 35	Spontaneous AP	Dexamethasone IV 1 mg/kg q6h for 3 days	NPO, IV fluids, analgesia, proton pump inhibitors, parenteral nutrition, modified Dachengqi Decoction, plasma and albumin if necessary, 14d antimicrobials	Observation and follow up for 1 month	NA	NA	Duration of hospitalisation significantly reduced in GC group (P = 0.03) 32.5 vs 40.2 days	GC group: 8.6%, model controls: 14.3% (NS)	NA
Zhang et al. [41]*	LOE II RCT, moderate estimated risk of bias, moderate number of well characterised rats in each group	Rats	Model controls: 45 GC group: 45	Induced, sodium taurocholate 3.5% 1 mL/kg injected into bile-pancreatic duct	Dexamethasone 5 mg/kg IV single dose, 15 min post induction	None stated	Survival at 3,6,12 h, pancreas histopathology score 0–4 (oedema, acinar necrosis, inflammation, perivascular infiltrate, haemorrhage, fat necrosis)	NA	NA	NA	No difference in mortality	Pancreas histopathology score lower in GC group vs model controls at 3 and 6 h (P < 0.05), and at 12 h (P < 0.01)
Schulz et al. [42]	LOE II RCT, low estimated risk of bias, small number of well characterised rats in each group	Rats	Model controls: 15 GC group: 15	Induced AP, sodium taurocholate 3.5% 2 mL/kg injected into the biliary-pancreatic duct	Methylprednisolone 25 mg/kg IV, 1 h post induction, followed by CRI 0.125 mg/kg	Ringers lactate 0.5 mL/h	Survival, histopathology at 20 h score 0–3 (oedema, inflammatory infiltration, haemorrhage, necrosis, fat necrosis)	NA	NA	NA	No difference in survival	No difference in histopathology
Gloor et al. [61] Experiment 1	LOE III Controlled trial, low estimated risk of bias, moderate number of well characterised rats in each group	Rats	Model controls: 32 GC group: 32	Induced AP, cerulein 5 µg/kg/h over 6 h IV (oedematous pancreatitis)	Hydrocortisone 10 mg/kg IV 10 min post induction	Fluid replacement	Histopathology at 1.5 h, 3 h, 6 h, 12 h; score 0–4 (oedema, inflammatory infiltration), 0–7 (necrosis, haemorrhage)	NA	NA	NA	NA	No difference in histopathology score, results not shown
Gloor et al. [61] Experiment 2	LOE III Controlled trial, low estimated risk of bias, moderate number of well characterised rats in each group	Rats	Model controls: 40 GC group: 40	Induced AP, sodium taurocholate 5% injected into the biliopancreatic duct (necrotising pancreatitis)	Hydrocortisone 10 mg/kg IV 10 min post induction	Fluid replacement	Histopathology at 1.5 h, 3 h, 6 h, 12 h; score 0–4 (oedema, inflammatory infiltration), 0–7 (necrosis, haemorrhage)	NA	NA	NA	NA	No difference in histopathology score, results not shown
Gloor et al. [61] Experiment 3	LOE III Controlled trial, low estimated risk of bias, very small number of well characterised rats in each group	Rats	Model controls: 8 GC group: 21	Induced, sodium taurocholate 5% injected into the biliopancreatic duct (necrotising pancreatitis)	Hydrocortisone 10 mg/kg IV 10 min post induction	Fluid replacement	72 h survival	NA	NA	NA	Reduced in GC group versus model controls (P = 0.01)	NA
Cosen-Binker et al. [43]	LOE II RCT, moderate estimated risk of bias, small number of well characterised rats in each group	Rats	Model controls: 16 Group 1: 16 Group 2: 16	Induced AP, sodium taurocholate 7% injected into the biliopancreatic duct	Hydrocortisone SC 4 h post induction Model controls: No GC Group 1: GC 4 mg/kg Group 2: GC 2 mg/kg	None stated	Histopathology at 8 h post induction, score 0–4 (oedema, haemorrhage, leucocyte infiltration, acinar necrosis, fat necrosis)	NA	NA	NA	NA	Unclear description of results numerical and statistical differences were found
Cosen-Binker et al. [44]	LOE II RCT, low estimated risk of bias, small number of well characterised rats in each group	Rats	Model controls: 16 Group 1: 16 Group 2: 16 Group 3: 16 Group 4: 15	Induced AP, sodium taurocholate 8% 1 mL injected into the biliopancreatic duct	Hydrocortisone 6 mg/kg SC or Prednisolone 0.5 mg/kg SC 1 h or 4 h post induction Controls: No GC Group 1: HC 1 h Group 2: HC 4 h Group 3: Pred 1 h Group 4: Pred 4 h	None stated	CRP, histopathology at 10 h, score 0–4 (oedema, haemorrhage, leukocyte infiltration, acinar necrosis, fat necrosis)	NA	HC or pred 1 h post induction improvement of CRP (P < 0.05) HC or pred 4 h post induction no difference	NA	NA	HC or pred 1 h post induction improvement of histopathology score (P < 0.05). HC or pred 4 h post induction no difference
Jha et al. [45]	LOE II RCT, high estimated risk of bias, moderate number of well characterised rats in each group	Rats	Model controls: 24 GC group: 24	Induced AP, sodium taurocholate 4% 1 mL/kg injected into the biliopancreatic duct	Dexamethasone 0.5 mg/kg IV after induction	None stated	Histopathology at 3 h, 6 h and 12 h, score 0–4 (oedema, acinar necrosis, haemorrhage, fat necrosis, inflammation, perivascular infiltration), TEM of pancreatic tissue	NA	NA	NA	NA	Authors claim marked difference in histopathology and TEM pathologic changes between groups at 12 h Results not shown
Melo et al. [46]	LOE III RCT, low estimated risk of bias, very small number of well characterised rats in each group	Rats	Model controls: 8 GC group: 8	Induced AP, l-arginine 2.5 g/kg IP twice with 1 h interval	Methylprednisolone 30 mg/kg PO 1 h after induction	None stated	Histopathology at 24 h, score 0–3 (oedema, leukocyte infiltration, haemorrhage, acinar vacuolization & necrosis)	NA	NA	NA	NA	Reduced histopathology score on all parameters in GC group compared to model controls (P < 0.05)
Ramudo et al. [62]	LOE III Controlled trial, moderate estimated risk of bias, very small number of well characterised rats in each group	Rats	Model controls: 6 GC group: 6	Induced AP, bile-pancreatic duct obstruction (BPDO)	Dexamethasone 1 mg/kg IM 1 h post induction	Buprenorphine 0.2 mg/kg IM	Histopathology at 3 h and 12 h, score 0–3 (oedema, inflammatory cells, vacuolisation, necrosis)	NA	NA	NA	NA	Reduced oedema and leucocyte infiltration at 12 h in GC group compared to model controls (P < 0.05)
Ramudo et al. [47]	LOE II RCT, low estimated risk of bias, unknown number of well characterised rats in each group	Rats	Model controls: not stated GC group: not stated	Induced AP, sodium taurocholate 3.5% 0,1 mL injected into the biliopancreatic duct	Dexamethasone 1 mg/kg IM 1 h post induction	Buprenorphine 0.2 mg/kg IM	Histopathology at 3 h and 6 h, score 0–3 (oedema, inflammatory cells, vacuolisation, necrosis)	NA	NA	NA	NA	Reduced necrosis at 6 h in GC group compared to model controls (P < 0.05)
Ou et al. [48]**	LOE II RCT, high estimated risk of bias, moderate number of well characterised rats in each group	Rats	Model controls: 36 GC group: 36	Induced, sodium taurocholate 3.5% 0.1 mL/100 g injected into the biliopancreatic duct	Dexamethasone 0.5 mg/100 g IV 15 min post induction, then CRI 0.05 mg/100 g/h (dose only mentioned in Zhang et al. 2010)	None stated	Mortality rate, Pathological severity score at 3 h, 6 h, 12 h (not further explained), but results shown	NA	NA	NA	Lower in GC group compared to model controls at 12 h (P < 0.05)	Reduced histopathology severity score at 12 h in GC group compared to model controls (P < 0.05)
Zhao et al. [49]	LOE II RCT, high estimated risk of bias, small number of well characterised rats in each group	Rats	Model controls: 15 GC group: 15	Induced AP, sodium taurocholate 5% injected into the biliopancreatic duct	Dexamethasone 5 mg/kg IV following induction	None stated	Histopathology, scoring technique not stated, results not shown	NA	NA	NA	NA	Author claims Oedema and necrosis reduced in GC group compared to model controls especially at 12 h
Foitzik et al. [50]	LOE II RCT, moderate estimated risk of bias, small number of well characterised rats in each group	Rats	Model controls: 10 Group 1: 10 Group 2: 10 Group 3: 12	Induced AP: Glycodeoxycholic acid 10 mM injected into the biliopancreatic duct, then IV 5 µg/kg/h cerulein over 6 h	Prednisolone started 6 h after induction, q8h for 24 h Ccontrols: No GC Group 1: GC 2 mg/kg/day Group 2: GC 10 mg/kg/day Group 3: GC 50 mg/kg/day	IV fluids 6 mL/kg/h for 8 h after induction	Mortality, histopathology 6 h after last treatment, score 0–4 (oedema, acinar necrosis, haemorrhage, fat necrosis, inflammation, perivascular infiltrate)	NA	NA	NA	Mortality 20–40%, no difference among groups	Histopathology score for acinar necrosis: No difference among groups
Biradar and Veeresh [51]	LOE II RCT, high estimated risk of bias, very small number of well characterised rats in each group	Rats	Model controls:6 GC group: 6	Induced: 2 Intraperitoneal injections of l-arginine 2.5 g/kg, 1 h apart	Methylprednisolone 30 mg/kg/day PO 1 h post induction	None stated	CRP histopathology 24 h after last L-arginine injection, score 0–3 (oedema, acinar cell degeneration, interstitial inflammation, haemorrhage),	NA	CRP results for GC group not show	NA	NA	Authors claim less oedema, Inflammation, acinar cell degeneration & necrosis in GC group, results not shown
Biradar and Veeresh [52]	LOE II RCT, high estimated risk of bias, very small number of well characterised rats in each group	Rats	Model controls: 8 GC group: 8	Induced: 2 Intraperitoneal injections of l-arginine 2.5 g/kg, 1 h apart	Methylprednisolone 30 mg/kg/day PO 1 h post induction	None stated	CRP, histopathology 24 h after last L-arginine injection, score 0–3 (oedema, acinar cell degeneration, interstitial inflammation, haemorrhage)	NA	CRP results for GC group not shown	NA	NA	Authors claim less oedema, Inflammation, acinar cell degeneration & necrosis in GC group, results not shown
Duan et al. [63]	LOE III Controlled trial, moderate estimated risk of bias, moderate number of well characterised rats in each group	Rats	Model controls: 24 GC group: 21	Induced AP: sodium taurocholate 5% 1 mL/kg injected into the intracholangiopancreatic duct	Methylprednisolone 30 mg/kg IV 30 min after induction	6 mL/kg/h SC saline	Mortality	NA	NA	NA	4/24 model controls died, 0/21 in GC group died	NA
Wang et al. [36]	LOE II RCT, moderate estimated risk of bias, moderate number of well characterised rats in each group	Rats	Model controls: 25 GC group: 25	Induced AP: sodium taurocholate 5% 1 mL/kg injected into the intracholangiopancreatic duct	Dexamethasone 0.5 mg/kg IV 5 min after induction	None stated	Mortality, histopathology at 12 h, score 0–4 (oedema, acinar necrosis, haemorrhage, fat necrosis, inflammation, perivascular infiltrate)	NA	NA	NA	Model controls: 42.9% GC group: 0% (P < 0.01)	Haemorrhage & acinar necrosis less in GC group compared to model controls (P < 0.05) Results not shown
Liu et al. [53]	LOE II RCT, low estimated risk of bias, small number of fairly well characterised rats in each group	Rats	Model controls: 16 GC group: 16	Induced AP: sodium taurocholate 5% 0.4 mL/kg injected into the intracholangiopancreatic duct	Glucocorticoid (type not stated) 20 mg/kg SC 1 h after induction	None stated	Mortality 24 h postinduction, histopathology at 3 h, 6 h and 12 h, score 0–3 (oedema, acinar necrosis, inflammatory infiltrate, haemorrhage, fat necrosis, perivascular inflammation)	NA	NA	NA	Model controls 50% GC group 40%, Not significant	Histopathology scores were lower in the GC group compared to model controls (P < 0.05)
Sha et al. [54]	LOE II RCT, low estimated risk of bias, moderate number of well characterised rats in each group	Rats	Model controls: 24 GC group: 24	Induced AP: Taurocholate 40 g/L 1 mL/kg injected into the Intracholangiopancreatic duct	Dexamethasone 0.5 mg/kg IV after induction	None stated	Histopathology score 0–3 (oedema, acinar necrosis, haemorrhage, fat necrosis, inflammation, perivascular infiltrate and TEM at 3 h, 6 h and 12 h	NA	NA	NA	NA	Histopathologic scores lower in GC group compared to model controls (P < 0.05) Ultrastructural changes were markedly less in the GC group
Kilic et al. [55]	LOE II RCT, low estimated risk of bias, very small number of well characterised rats in each group	Rats	Model controls: 8 GC group: 8	Induced AP: cerulein injected IP hourly 5 times, given a total of 80 mg/kg	Methylprednisolone 10 mg/kg IM twice hourly at 1 h after last cerulein injection	None stated	Histopathology at 20 h, score 0–3 (oedema), score 0–4 (inflammation, necrosis, vacuolisation)	NA	NA	NA	NA	Histopathology scores lower in GC group compared to model controls (P < 0.01)
Cui et al. [56]	LOE II RCT, high estimated risk of bias, very small number of well characterised rats in each group	Rats	Model controls: 6 GC group: 6	Induced AP: sodium taurocholate 5% 1 mL/kg injected into intracholangiopancreatic duct	Dexamethasone 1 mg/kg IP 3 h post induction	None stated	Histopathology at 24 h post treatment, no technique or results shown, only photos	NA	NA	NA	NA	Histopathological changes were alleviated in GC group according to authors
Zhao et al. [64] Group 1	LOE III Controlled trial, high estimated risk of bias, small number of well characterised mice in each group	Mice C57BL/6	Model controls: 34 Group 1: 20 Group 2: 15	Induced: l-arginine 2.5 mg/g injected IP twice with 1 h interval	Dexamethasone 30 min post induction controls: No GC Group 1: GC 0.4 mg/kg IV Group 2: GC 4 mg/kg IV	Geldanamycin 1 or 10 g/kg IV 10 min post induction	Mortality at 24 h, histopathology at 24 h, no technique or results shown, only photos	NA	NA	NA	Model controls: 23.5% Group 1: 15% Group 2: 6.7%	Pictures of histopathology are shown but not discussed
Zhao et al. [64] Group 2	LOE III Controlled trial, moderate estimated risk of bias, small number of well characterised mice in each group	Mice BALB/c	Model controls: 34 Group 1: 20 Group 2: 15	Induced: l-arginine 2.5 mg/g injected IP twice with 1 h interval	Dexamethasone 0.4 or 4 mg/kg IV 30 min post induction controls: No GC Group 1: GC 0.4 mg/kg IV Group 2: GC 4 mg/kg IV	Geldanamycin 1 or 10 g/kg IV 10 min post induction	Mortality at 24 h, histopathology at 24 h, no technique or results shown, only photos	NA	NA	NA	Model controls: 52.9% Group 1: 45% Group 2: 6.7%	Pictures of histopathology are shown but not discussed

Model controls: AP cases receiving no or standard (other) treatment, GC group: AP cases treated with GC in addition to treatment for model controls

^*^10 articles were found describing the same experimental study, the most relevant was used as reference here, the other 9 was full text excluded, see Fig. 1, a reference list is available at request

^**^2 articles were found describing the same experimental study, the most relevant was used as reference here, the other was full text excluded, see Fig. 1, a reference list is available at request

AP: acute pancreatitis; CRI: constant rate infusion; CRP: C-reactive protein; GC: glucocorticoid; h: hours; HC: hydrocortisone; IM: intramuscular; IV: intravenous; IP: intraperitoneal; NA: not applicable; NPO: nothing per os; Pred: Prednisolone; RCT: randomised controlled trial; SC: subcutaneous; TEM: transmission electron microscopy

### Data item

The following information was assessed or directly extracted from each included study: (1) Level of evidence, study design and methodological quality; (2) Species studied; (3) Number of individuals in corticosteroid-treated groups (GC groups) and model control groups; (4) Type of acute pancreatitis (AP) (spontaneous or experimentally induced); (5) Corticosteroid treatment; (6) Other treatments; (7) Data collection and timing; (8) Outcome: Clinical score (as defined by the authors); (9) Outcome: CRP level; (10) Outcome: Hospitalisation duration; (11) Outcome: Mortality rate; and (12) Outcome: Pancreas histopathology score.

### Critical appraisal of individual sources of evidence

Both authors individually assessed the level of evidence and quality of the method for all included studies. In case of discrepancies, a consensus was reached through discussion.

The individual studies were graded for level of evidence (LOE) on a scale of I to IV [30]. LOE I studies included relevant primary studies (systematic reviews and meta-analyses were originally included but were ultimately excluded) (Fig. 1); LOE II studies included randomised controlled trials (RCTs); LOE III studies included nonrandomised controlled trials and retrospective case–control series; and LOE IV studies included case series and represented the lowest LOE.

The quality of the method of the individual studies was evaluated for the following 3 components: group similarity, risk of bias, and study group sizes.

Group similarity was rated good, fair or poor according to the description of health/disease status, age (or weight of rodents), sex and breed (dog studies only) of study subjects. When experimental animals were used, no description of disease was interpreted to mean that the animals were healthy.

Good group similarity: All healthy before induction of pancreatitis, or acute pancreatitis (AP) diagnoses for all participants based on clinical signs compatible with AP and at least one of the following: Positive cPLI (Spec cPL) or DGGR lipase, diagnostic imaging (ultrasound or CT) findings indicative of AP, histopathological findings indicative of AP, and similar age, sex and breed characteristics.

Fair: All healthy or AP diagnosis for all participants based on at least clinical signs compatible with AP and increased plasma lipase/amylase, no obvious skewing of group participants regarding age, sex and breed.

Poor: Information on diagnostic criteria for AP diagnosis is lacking or information regarding age/weight, breed or sex of study subjects is lacking.

The risk of bias of individual studies was graded as high, moderate or low according to the evaluated risk of selection, performance, detection, attrition, reporting and other bias. The evaluation was performed using the criteria set up by the Cochrane Collaboration’s tool for assessment of risk of bias [31].

Study group size was graded as good, moderate, small or very small according to criteria used in other veterinary systematic reviews: > 50 (good), 20–49 (moderate), 10–19 (small), < 10 (very small) [32–35]. The collected information is presented in Table 2.

**Table 2:** Risk of bias in individual studies evaluating the effect of glucocorticosteroids for acute pancreatitis

Study	Study species	Study design	LOE I–IV	Group similarity (good, fair, poor)	Estimated risk of bias (high, moderate, low)	Support for Risk of bias judgement	Group size
Okanishi et al. [57]	Dogs	Non-randomised controlled trial	III	Good (age, sex, breed, diagnosis incl clinical signs + spec-cPLI or plasma lipase, ultrasound, CRP. Baseline variables were similar between groups	Moderate	Selection bias: Non-concealed, non-randomised allocation of participants to GC group 2011–2014, non-GC group 2015–2016. Breeds and ages in groups were not matched. Performance bias: No blinding. V-LIP used in diagnosis is inferior to spec-cPLI. Detection bias: No blinding of outcome assessment. Statistical analysis described. 3 drop-outs excluded from relevant analysis	Moderate (45/20)
Studley and Schenk [39] Experiment 1 and 2	Dogs	Randomised controlled trial	II	Poor, age/sex/breed not stated. Diagnosis ok as histopathology confirmed induced pancreatitis	Low	Selection bias: Method of randomisation not described. Performance bias: No blinding, detection bias: No blinding of outcome assessment, but as outcome was survival, the lack of blinding was less worrying. No drop-outs. Stat analysis, but not described	Very small (5–8)
Attix et al. [58]	Dogs	Controlled trial	III	Poor, age not stated, division of which sex into which group not stated, all “mixed-breed”, induced AP diagnosis was confirmed by histopathology	High	Selection bias: No randomisation/concealment. Performance bias: No blinding of personnel performing subjective clinical evaluation and histopathology. Detection bias: no blinding of outcome assessment. Reporting bias: clinical and pathologic features were to be compared for GC treated and non-GC treated, no results for histopathology were reported. No drop-outs, no statistics	Very small (4/4)
Stewart et al. [59]	Dogs	Controlled trial	III	Poor, age not stated, division of which sex into which group not stated, all “mixed-breed”, induced AP diagnosis was confirmed by histopathology	High	Selection bias: no randomisation/concealment. Performance: No blinding. Detection bias: No blinding of outcome assessment, however, survival is an objective parameter, histopathology less so. 3 cortisone treated died of GI ulceration biasing the mortality rates. No drop-outs, no statistics	Moderate/small (25/16)
Imahori et al. [60]	Dogs	Controlled trial	III	Poor, age and sex not stated, all “mixed-breed”, induced AP diagnosis was confirmed by histopathology	Low (High for electron microscopy)	Selection bias: No randomisation/concealment. Blinded histopathologist. No drop-outs, statistics but not explained	Study 1 small (14/14), study 2 very small (5/6)
Kaplan et al. [65]	Humans	Retrospective case–control study	III	Fair, AP diagnosis by clinical signs and plasma amylase, age and sex stated	High	Selection bias: No randomisation/concealment. No standardisation among groups in diagnostics or treatments. Performance bias: No blinding of personnel or participants. Variation in dose and timing of GC and other treatment. High incidence of gall bladder disease. Detection bias: no blinding of outcome assessment. No drop-outs, no statistics	Small (15/48)
Liu et al. [40]	Humans	Randomised controlled trial	II	Poor, AP diagnosis by clinical diagnosis (not further elaborated), age and sex of patients not mentioned	Moderate	Selection bias: Method of randomisation not described. Detection bias: Lack of blinding of outcome assessment seems irrelevant, as outcome is mortality. Other bias: Salviae Miltiorrhiza and Dextran was given with GC. In 2 of the 3 deaths in GC group, early preventive treatment was delayed till 3 days after onset of disease. No drop-outs, statistics explained	Moderate (26/53)
Eklund et al. [37]	Humans	Retrospective case–control study	III	Good, AP diagnosis by clinical signs, amylase and CT, age and sex distribution similar among groups	Low	Selection bias: Only patients with severe acute pancreatitis requiring norepinephrine support for hemodynamic shock were included. Performance bias and detection bias: No blinding, but outcomes CRP and mortality are objective parameters. Other comments: Cause of AP: Alcohol 7, gallstones 3, unusual causes for dogs, should be considered when results are extrapolated for dogs. No drop-outs, statistics	Small (10/11)
Wang et al. [66]	Humans	Case-series	IV	Fair: AP diagnosis by clinical signs, amylase and ultrasound, CT, age and sex stated	High	Performance and detection bias: No blinding of participants or personnel, who evaluated pain. Reporting bias: Comparison of mortality with literature is questionable. Other bias: Dextran 40 was given along with GC. No drop-outs mentioned, no statistics	Moderate (32)
Wan et al. [38]	Humans	Randomised controlled trial	II	Good, AP diagnosis by clinical signs, lipase, CT, no statistical difference among groups according to sex, age, disease severity, etiology, complications	Low	Randomisation by SPSS software, drop-out rate not statistically significant different between groups, Performance and detection bias: no blinding stated but all parameters objective. Statistics explained	Moderate (43/38)
Zhang et al. [41]	Rats	Randomised controlled trial	II	Good, healthy, all male, similar weight rats	Moderate	Selection bias: Method of randomisation not described. Performance and detection bias: No blinding of histopathologist stated, but semiquantative grading of histopathologic findings. Attrition bias: histopathology score of 2 rats from control group was not available due to their death at 12 h. No other drop-outs, statistics explained	Moderate (45/45)
Schulz et al. [42]	Rats	Randomised controlled trial	II	Good, all male, similar weight rats	Low	Selection bias: Method of randomisation not described. Blinded histopathologist, semiquantative grading of histopathology findings, no drop-outs, statistics explained	Small (15/15)
Gloor et al. [61]	Rats	Controlled trials	III	Good, all female, similar weight rats	Low	Selection bias: No randomisation stated, blinded histopathologists, semiquantative grading of histopathology findings, no drop-outs, no statistics on histopathology, but statistics on mortality	Moderate (32/32) Moderate (40/40) Moderate/very small (21/8)
Cosen-Binker et al. [43]	Rats	Randomised controlled trial	II	Good, pathogen-free, all male similar weight rats	Moderate	Selection bias: Method of randomisation not described. Blinded histopathologists, semiquantative grading of histopathological findings, no drop-outs. Reporting bias: Unclear description of results: GC “did not present much improvement”, although results are marked by P < 0,001. Results and statistics not adequately explained	Small (16/16/16)
Cosen-Binker et al. [44]	Rats	Randomised controlled trial	II	Good, pathogen-free, all male similar weight rats	Low	Selection bias: Method of randomisation not described. Blinded histopathologists, semiquantative grading of histopathology findings, no drop-outs. Reporting bias: Authors state that treatment 4 h post induction is harmful, but results put these in the same disease category as controls. Statistics	Small (16/16/16/16)
Jha et al. [45]	Rats	Randomised controlled trial	II	Good, all male similar weight rats	High	Selection bias: Method of randomisation not described. Blinded histopathologist. Reporting bias: No results of histopathology score or TEM shown, only photo examples with conclusions, no statistics on histopathology score of GC group or TEM. No drop-outs	Moderate (24/24)
Melo et al. [46]	Rats	Randomised controlled trial	III	Good, all male similar weight rats	Low	Selection bias: Randomisation not stated. Blinded histopathologist, semiquantative grading of histopathology findings, no drop-outs, statistics	Very small (8/8)
Ramudo et al. [62]	Rats	Controlled trial	III	Good, all male similar weight rats	Moderate	Selection bias: No randomisation stated. Blinded histopathologists, semiquantative grading of histopathology findings, no drop-outs, statistics	Very small (6/6)
Ramudo et al. [47]	Rats	Randomised controlled trial	II	Good, all male similar weight rats	Low	Selection bias: Method of randomisation not described. Blinded histopathologists, semiquantative grading of histopathology findings, no drop-outs, statistics	Unknown, group sizes not stated
Ou et al. [48]	Rats	Randomised controlled trial	II	Good, clean grade, all male similar weight rats	High	Selection bias: Method of randomisation not described. Performance and detection bias: Blinding not stated. Reporting bias: Results of “pathological severity score” are concluded upon, but their definition is not explained. No drop-outs, statistics	Moderate (36/36)
Zhao et al. [49]	Rats	Randomised controlled trial	II	Good, all male similar weight and age	High	Selection bias: Method of randomisation not described. Performance, detection and reporting bias: Blinding of histopathologist(s) was not stated, no scoring technique described, histopathology results not shown, but conclusions were stated. No research question about histopathology, no statistics on histopathology, no drop-outs	Small (15/15)
Foitzik et al. [50]	Rats	Randomised controlled trial	II	Good, all male similar weight rats	Moderate	Selection bias: Method of randomisation not described. Performance and detection bias: Blinding of histopathologist(s) was not stated, semiquantitative histopathology scoring was done, Possible attrition bias: histopathological analysis included only those animals that survived the whole experiment. Statistics	Small (10/10/12/10)
Biradar and Veeresh 2012 [51]	Rats	Randomised controlled trial	II	Good, all male similar weight rats	High	Selection bias: Method of randomisation not described. Blinded histopathologist. Reporting bias: GC not in the research question, used as positive control, no results or conclusion for CRP shown, no results for histopathology score shown, but conclusions stated. No drop-outs, no statistics	Very small (6/6)
Biradar and Veeresh [52]	Rats	Randomised controlled trial	II	Good, all male similar weight rats	High	Selection bias: Method of randomisation not described. Blinded histopathologist. Reporting bias: GC not in the research question, used as pos control, no results or conclusion for CRP shown, no results for histopathology score shown, but conclusions stated. No drop-outs, no statistics	Very small (8/8)
Duan et al. [63]	Rats	Controlled trial	III	Good, all male similar weight and age rats	Moderate	Selection bias: Randomisation not stated, only relevant parameter was mortality, which makes the lack of blinding less worrying. No drop-outs, no statistics	Moderate (21/24)
Wang et al. [36]	Rats	Randomised controlled trial	II	Good, all male similar weight rats	Moderate	Selection bias: Method of randomisation not described. Blinded histopathologists. Reporting bias: Results for histopathology were not shown, but concluded upon. No drop-outs, statistics	Moderate (25/25)
Liu et al. [53]	Rats	Randomised controlled trial	II	Fair, rats of similar weight, otherwise not described	Low	Selection bias: Method of randomisation not described. Blinded histopathologists. Performance bias: The type of glucocorticoid was not specified making the dose hard to evaluate. No drop-outs, statistics	Small (16/16)
Sha et al. [54]	Rats	Randomised controlled trial	II	Good, all male similar weight rats	Low	Selection bias: Method of randomisation not described. Blinded histopathologist. Reporting bias: Results are shown for histopathology score and there are conclusions and statistical analysis. For TEM no results are shown, but conclusions are made. No drop-outs	Moderate (24/24)
Kilic et al. [55]	Rats	Randomised controlled trial	II	Good, rats aged 7–8 months, similar weight	Low	Selection bias: Method of randomisation not described. Blinding, histopathology scoring system, clear results and statistical evaluation, research question was answered. No drop-outs	Very small (8/8)
Cui et al. [56]	Rats	Randomised controlled trial	II	Good, all male, similar age and weight	High	Selection bias: Method of randomisation not described. Performance, detection and reporting bias: No blinding, no stated histopathology scoring system, no statistical evaluation, seems to be conclusions based on subjective evaluation. No drop-outs	Very small (6/6)
Zhao et al. [64]	Mice	Controlled trial	III	Good, all male, similar age mice of 2 genotypes	High	Selection bias: No randomisation stated. Performance, detection and reporting bias: No blinding stated, histopathological changes of the pancreas was not included in the aims, but shown by photos and not commented on. Mortality rate was included in the aims, results provided, but no statistical evaluation was done. No drop-outs	Small (15/20/34)

The table displays the study designs and the estimated risk of bias in individual studies referred to in the review. The individual studies were graded for level of evidence (LOE) on a scale of I to IV, the group similarity was rated good, fair or poor and study group size was graded as good, moderate, small or very small

### Synthesis of results

The outcome parameters for evaluating the effect of corticosteroid treatment on acute pancreatitis were the clinical score (as defined by the authors), circulating CRP levels, hospitalisation duration, mortality rate and pancreas histopathology score. Among the included studies, evidence for the corticosteroid treatment effect for each outcome parameter was collected and summarised narratively. The evidence for each outcome parameter was graded good, fair or insufficient, according to the following criteria modified from Jensen and Bjørnvad [35]:

Good evidence: Multiple RCTs with a low risk of bias or with a moderate risk of bias and a moderate to good study size.

Fair evidence: At least 1 RCT had a low risk of bias or a moderate risk of bias and a moderate to good study size.

Insufficient evidence: No RCTs had a low risk of bias or a moderate risk of bias and moderate to good study size.

Good or fair evidence for positive effect of corticosteroid treatment would indicate support of corticosteroid treatment. Alternatively, good or fair evidence for lack of effect or adverse effects would result in the advice against corticosteroid treatment.

### Ethical considerations

In 20 of the 21 experimental rodent studies, the study protocols were documented to have followed national ethical guidelines and/or had been approved by an ethical committee; the exception was Wang et al. [36]. No ethical considerations were mentioned in the canine studies. Two human studies were approved by an ethical committee [37, 38].

## Review

### Study selection

After removing duplicates and non-English language articles, a total of 2230 citations were identified by electronic databases and manual searches of article references. Screening of titles and abstracts excluded 2170 of these citations. Sixty full-text articles were assessed for eligibility, and 29 were excluded for reasons stated in the PRISMA flow diagram (Fig. 1). A reference list of the 29 excluded studies is available on request. Finally, 31 studies were included in this review.

### Study characteristics

Of the 31 studies included in the review, 5 involved dogs, 5 involved humans and 21 involved rodents (Table 1).

Regarding the study design, 20 studies were categorised as RCTs (Tables 1 and 2); of these, 1 was a canine study [39], 2 were human studies [38, 40] and 17 were rodent studies [36, 41–56]. Only 1 study [38] described the method of randomisation. Eight studies were categorised as nonrandomised controlled trials, with 4 involving canines [57–60] and 4 involving rodents [61–64]. Two human studies were categorised as retrospective case control studies [37, 65], and 1 human study was categorised as a case series [66] (Tables 1 and 2).

The studies involved mainly very small to moderate size study groups ranging between 4 and 45 participants (dogs, humans and rodents). One control group had 53 participants [40] (Table 2).

One canine study concerned dogs with spontaneously occurring AP [57], and 4 studies concerned healthy dogs with experimentally induced AP [39, 58–60]. The 5 human studies all concerned patients with spontaneously occurring AP receiving treatment in hospital settings (Table 1). All 21 rodent studies concerned experimentally induced AP. Methods of inducing experimental AP varied among the studies (Table 1).

All subjects in the included studies were provided similar supportive treatment. Dogs suffering from spontaneous AP were treated according to standard protocols, including IV fluids, antibiotics, opioid analgesics, antiemetics, antacids and vitamin supplements [57]. Human subjects were treated with the previously mentioned supportive treatments as well as anticholinergics, nasogastric suction, pancreatin secretion inhibition, parenteral nutrition, oxygen, dextran 40, Chinese Dachengqi decoction, and blood plasma and albumin transfusion [37, 38, 40, 65, 66]. Rodents and dogs subjected to induced AP were generally not treated or only treated with IV or SC fluids. Few were treated with analgesics [47, 62] or antimicrobials [58, 64]. In addition, test subjects (GC groups) were given corticosteroids while model controls were not.

Different types of corticosteroids were used in the included studies. Seven studies used hydrocortisone, including 2 dog studies [39, 60], 2 human studies [37, 65] and 3 rodent studies [43, 44, 61]. Prednisolone was used in 1 canine study [57] and 2 rodent studies [44, 50], while methylprednisolone was used in 6 rodent studies [42, 46, 51, 52, 55, 63]. Cortisone acetate was used in just 1 canine study [59], whereas dexamethasone was used in 1 canine study [58], 3 human studies [38, 40, 66] and 10 rodent studies [36, 41, 45, 47–49, 54, 56, 62, 64]. The type of corticosteroid used for treatment was not specified in one of the rodent studies [53]. The dose, administration route and start time of corticosteroid treatment varied widely among studies (Table 1).

Of the outcome parameters, the clinical score was reported in 4 studies. In dogs, Attix et al. [58] subjectively evaluated the activity and general appearance by clinical examination while Okanishi et al. [57] scored patients 0–3 on specific parameters (weakness/lethargy, appetite, vomiting, stool condition, abdominal pain). In the two human studies, the clinical outcome evaluation method was not described [65, 66].

Circulating CRP was measured in 5 studies, namely, 1 canine [57], 1 human [37] and 3 rodent studies [44, 51, 52]; however, for 2 of the rodent studies [51, 52], the results were not presented.

The duration of hospitalisation was reported in 1 canine study [57] and 1 human study [38].

Mortality rates were reported in 18 studies, including 4 canine [39, 57, 59, 60], all 5 human studies [37, 38, 40, 65, 66] and 9 rodent studies [36, 41, 42, 48, 50, 53, 61, 63, 64].

Histopathological examination of the pancreas was performed in 23 studies, including 3 canine and 20 rodent studies (Table 1). A histopathological scoring system was used in 17 of these 23 studies. Oedema, inflammatory infiltration, necrosis and haemorrhage was frequently rated using a score of 0–3 or 0–4, sometimes, fat necrosis or vacuolisation was rated in a similar way. In 6 studies, the histopathological scoring technique was not reported [48, 49, 56, 58, 59, 64]. Ten studies did not present histopathological results [36, 49, 51, 52, 56, 58, 59, 61, 64]; of these, 7 studies reported conclusions that were not statistically evaluated [45, 49, 51, 52, 56, 59, 61] while 1 reported statistically significant conclusions [36]. In one study the results were considered unclear [43].

### Risk of bias within studies

An overview of the “risk of bias” for the individual studies can be found in Table 2. In general, a great variance in risk of bias was found among the studies, and it did not seem to depend on time since publication.

For studies concerning spontaneously occurring AP, the estimated risk of bias was categorised as low for 2 human studies [37, 38], moderate for 1 canine study [57] and 1 human study [40], and high for 2 human studies [65, 66]. Study group sizes were found to be moderate in 1 canine study [57] and 3 human studies [38, 40, 66] and small in 2 human studies [37, 65]. Similarity at baseline was poorly characterised in 1 human study [40], fair in 2 human studies [65, 66] and good in 1 canine study [57] and 2 human studies [37, 38].

For studies concerning dogs with experimentally induced AP, the estimated risk of bias was categorised as low for 2 studies [39, 60] and high for 2 studies [58, 59]. Study group sizes were small or very small, and similarity at baseline was poorly characterised for all 4 studies [39, 58–60].

Studies concerning rodents with experimentally induced AP varied in risk of bias. A low risk of bias was found in 8 studies [42, 44, 46, 47, 53–55, 61]. Six studies were categorised as moderate [36, 41, 43, 50, 62, 63], and 7 studies were categorised as high [45, 48, 49, 51, 52, 56, 64]. Regarding group size, 6 studies were categorised as very small [46, 51, 52, 55, 56, 62], 7 studies were categorised as small [42–44, 49, 50, 53, 64], 7 studies were categorised as moderate [36, 41, 45, 48, 54, 61, 63] and 1 study did not provide group size information [47]. With regard to similarity at baseline, all studies except for 1 were categorised as well characterised, and the exception was categorised as fair [53].

No studies had sample size calculations performed prior to the start of the study.

Few studies mentioned drop-outs. Okanishi et al. [57] had 3 drop-outs, which they excluded from relevant analyses without commenting on possible implications regarding the results. No other canine studies mentioned drop-outs. In human studies, only Wan et al. mentioned that statistically significant differences were not observed in the experimental groups following drop-outs, and they clearly described the drop-out cases [38]. In the rodent studies, one study chose to exclude from the histopathological analysis rodents that died during the experiment; numbers were not presented and possible implications for the results were not described [50]. The rest of the studies did not report drop-outs.

Several studies did not include statistical analyses. Among the human studies, 2 provided clinical score and mortality conclusions without presenting statistical analyses [65, 66]. Two canine studies did not present statistical evaluations of their conclusions on clinical score [58], mortality and histopathology [59]. Six rodent studies did not statistically evaluate histopathological outcomes [45, 49, 51, 52, 56, 61]. Two rodent studies presented conclusions on mortality without statistical analyses [63, 64].

### Results of the individual studies and synthesis of results

For an overview of the results of individual studies, please refer to Tables 1 and 2.

Clinical score results were reported in 4 studies. One canine study on spontaneous AP reported a significantly shortened time (median 4 versus 7 days) to reach a clinical severity score of ≤ 2/3 compared to the model controls [57]. Three other studies (1 canine and 2 human studies) reported results on clinical scores without statistically evaluating the data [58, 65, 66].

Regarding the effect of corticosteroids on circulating CRP levels, one rodent study [44] found that hydrocortisone or prednisolone administered 1 h after induction of experimental AP resulted in slight but statistically significant decreases in CRP levels (16.8 ± 1.4 and 19.2 ± 1.6 mg/dL, respectively) compared with the no-steroid treatment (25.6 ± 1.7 mg/dL, P < 0.05), while the same treatment administered 3 h later did not have these effects (24.3 ± 1.6 and 24.8 ± 1.8 mg/dL, respectively, Table 1). For spontaneously occurring AP in dogs, Okanishi et al. found that the time for CRP levels to reach < 2 mg/dL was significantly shorter in prednisolone-treated dogs than in model controls [57]. Eklund et al. [37] found that CRP levels were significantly lower in hydrocortisone-treated human patients at 48 h after starting treatment but not at 24 h relative to standard (non-corticosteroid)-treated patients (Table 1).

The duration of hospitalisation was evaluated in 1 canine and 1 human study [38, 57]. Both studies found that the duration of hospitalisation was significantly reduced (median 5 vs 8 days and 32.5 vs 49.2 days) in corticosteroid-treated patients compared to non-corticosteroid-treated patients (Table 1).

Eleven studies provided statistically evaluated results on the mortality rate (Table 1). Seven studies found a statistically significant improvement in survival or reduction in mortality. Four studies found no statistically significant difference in mortality/survival. In dogs with spontaneously occurring AP, Okanishi et al. [57] found that 1-month survival was significantly higher (88.7% vs 57.9%) in prednisolone-treated dogs than in dogs receiving no corticosteroids (Table 1). In humans, Liu et al. found significantly reduced mortality in dexamethasone-treated patients compared to non-corticosteroid-treated patients (Table 1) [40]. However, this study was confounded by the fact that the corticosteroid group was also treated with dextran and Salvia miltiorrhiza. Wan et al. [38] found no significant difference in mortality between dexamethasone-treated and standard-treated human patients (Table 1).

In canine studies of experimentally induced AP, two studies reported significantly increased survival time (Table 1). Studley and Schenk [39] administered low-dose hydrocortisone 6 or 12 h after induction of AP and administered hydrocortisone at low or high doses 6 h after induction, and both of these studies showed a significantly increased survival time with hydrocortisone treatment compared to IV fluids only, regardless of the dose or timing of treatment. Imahori et al. [60] administered a similar low dose of hydrocortisone 6 h after induction and found that survival time was significantly increased in the hydrocortisone-treated group compared to the non-corticosteroid-treated group.

Six rodent studies statistically evaluated the corticosteroid effects on mortality in experimentally induced AP (Table 1). Three studies found significantly improved survival [36, 48, 61], and 3 studies found no significant difference in survival between corticosteroid-treated and model controls [42, 50, 53]. A comparison of the studies highlighted certain features. Five out of 6 studies used the same mode of AP induction, and all studies reported high mortality rates of non-treated rodents (20–86%). However, the type, dose and timing of the corticosteroid treatment varied greatly. For the studies reporting no difference in mortality, a dose of 2, 10 and 50 mg/kg prednisolone [50], 25 mg/kg methylprednisolone [42] or 20 mg/kg glucocorticoid of unknown type [53] were used, and they were all administered 1 h or later following induction. For studies reporting significantly improved survival, rodents were treated with 10 mg/kg hydrocortisone [61], 5 mg/kg dexamethasone [48] or 0.5 mg/kg dexamethasone [36] within 15 min after induction.

Seven studies reported mortality results that were not statistically evaluated [37, 41, 59, 63–66]. Only the experimentally induced AP studies performed histopathological analyses of the pancreas (Table 1). Thirteen studies presented statistically evaluated results. Three of these did not report blinding of the histopathologists [41, 48, 50]. Eleven studies reported significantly less severe histopathological changes in the pancreas of corticosteroid-treated animal groups than in the noncorticosteroid-treated groups. Of these, 7 studies concluded that the pancreas histopathological score was significantly lower in the corticosteroid group than in the model control group. The results are generally based on similar scoring systems for oedema, acinar necrosis, inflammatory infiltrate, and haemorrhage and certain including rating of fat necrosis and perivascular inflammation as well [41, 44, 46, 53–55]. One of the studies did not define their grading system [48]. In 4 studies, the following histopathological changes were significantly milder than those in the control groups; acinar necrosis [60], oedema and leucocyte infiltration at 12 h [62]; necrosis at 6 h [47]; and haemorrhage and necrosis at 12 h [36]. Two studies found no significant difference between the histopathological findings in rodents treated with corticosteroids and untreated rodents [42, 50].

Three studies presented no histopathological results or unclear findings [43, 58, 64]. Seven studies did not present statistical evaluations of their results [45, 49, 51, 52, 56, 59, 61]. Furthermore, in 2 studies that presented electron microscopy results for the pancreas [45, 60], statistical evaluations were not performed.

### Risk of bias across studies

In the assessment of risk of bias that could influence the cumulative evidence, Performance and detection bias frequently contributed to a classification of moderate to high risk of bias (Table 2). Several studies reported no blinding of outcome assessment of subjective parameters, such as clinical score and histopathology [41, 48–50, 56, 58, 59, 65, 66].

Reporting bias had the potential to severely influence the cumulative evidence, especially in cases where histopathological examinations were performed without scoring guidelines, results were not reported or statistical evaluations of the results were not performed but conclusions were made nonetheless [36, 45, 48, 49, 51, 52, 56].

## Discussion

### Summary of evidence

The aim of the present review was to evaluate whether dogs with acute/acute-on-chronic pancreatitis that received standard treatment could benefit from additional treatment with corticosteroids to improve disease outcome as indicated by the clinical score, circulating CRP levels, hospitalisation duration, mortality rate and pancreas histopathologic evaluation.

For the clinical score, the overall evidence was insufficient. There were no RCTs with a low risk of bias or a moderate risk of bias and moderate study size to establish recommendations regarding the use of steroids to reduce clinical scores.

Regarding the circulating CRP levels, one RCT with a low estimated risk of bias [44] concluded that treatment within 1 h was significantly beneficial while treatment within 4 h was not. Hence, fair evidence was found that corticosteroid treatment can reduce CRP levels if given early in the disease process. Furthermore, a canine nonrandomised controlled trial and a human case control study found significant reductions in circulating CRP levels in patients treated with corticosteroids compared to the model controls [37, 57].

Fair evidence was found that corticosteroid treatment can shorten hospitalisation length. This evidence was based on 1 canine and 1 human study in which a significantly shortened duration of hospitalisation was reported in the corticosteroid-treated groups compared to the model controls [38, 57]. The latter was a RCT with low estimated risk of bias.

The overall evidence for corticosteroid treatment reducing mortality in AP is conflicting. Seven studies provided results of significantly improved survival of corticosteroid-treated patients compared to model controls. Of these, 3 studies were RCTs of low risk of bias or moderate risk of bias along with moderate study size, thus providing good evidence for improved survival with corticosteroid treatment of AP [36, 39, 40]. However, 4 studies found no significant difference in survival and all were RCTs presented a low estimated risk of bias or moderate risk of bias along with moderate study size [38, 41, 42, 53], thus providing good evidence that corticosteroid treatment is of no significant benefit for survival. Notably, in rodent studies with improved survival [36, 48, 61], corticosteroids were administered early in the experimentally induced disease process (within 15 min), whereas in most of the studies without improved survival [42, 50, 53], corticosteroids were given later in the disease process (1 h or later).

The overall evidence that corticosteroid treatment results in less severe histopathological changes in experimentally induced pancreatitis is good. Eight RCTs with low estimated risk of bias or moderate risk of bias that also had a moderate study size found significantly less severe histopathological changes in the pancreas in the corticosteroid-treated animal groups than in the model controls [36, 41, 44, 46, 47, 53–55]. One RCT with a low estimated risk of bias provided fair evidence that corticosteroid treatment results in no significant improvement of histopathology [42].

## Limitations

The most obvious limitation of this review is the limited number of studies performed in dogs. Therefore, the results are useful for providing general conclusions on acute pancreatitis but insufficient for generating robust conclusions on canine acute pancreatitis. Among the 5 canine studies included, only the work of Studley and Schenk [39] was a RCT with a low estimated risk of bias; thus, it was the only canine study with the power to grade evidence for the general results in this review. Nevertheless, 3 of the 4 other canine studies were consistent with the positive effect of corticosteroids reported by Studley and Schenk [39]. A controlled clinical trial of moderate group size was recently reported in Okanishi et al. [57], and they found a significant benefit of prednisolone treatment with regard to clinical score, CRP level, hospitalisation duration and survival in dogs. Stewart et al. [59] and Imahori et al. [60] both found a positive corticosteroid effect on survival and pancreas histopathology in dogs with experimentally induced pancreatitis. A striking difference between these studies and a previous canine study, in which corticosteroids did not confer a benefit [58], was the timing of corticosteroid treatment start. The above 4 studies started treatment early in the disease process (at diagnosis, or no later than 12 h after experimental induction) and the study by Attix et al. started treatment 24 h after induction [58].

Similarly, early treatment was supported in the included rodent studies. No positive effect of corticosteroid treatment on mortality was observed in studies that started treatment 1 h or later after induction, while a positive effect was frequently observed in studies that started treatment early (within 15 min). Cosen-Binker et al. [44] concluded that hydrocortisone or prednisolone administered 1 h after induction resulted in slight but significant improvement of histopathology score and CRP levels while the same treatments administered 3 h later did not. Overall, the findings indicate that the early start of corticosteroid treatment might be important for a positive effect.

Cosen-Binker et al. [44] indicated that hydrocortisone and prednisolone seemed to have the same effect, and Studley and Schenk [39] did not observe differences in the positive effect of low or high doses of hydrocortisone. In the included studies, a wide range of types and doses of corticosteroids were used, and identifying associations between the type or dose of corticosteroids and the effect was difficult. Any association found in this multispecies review would be of questionable use for specific canine treatment recommendations.

Another limitation of this review was that two of the human studies involved treatments with dextran or Salvia miltiorrhiza along with corticosteroids. Although these treatments are not recommended by international treatment protocols and could bias the results [40, 66], one of these studies was included here as evidence for reduced mortality [40].

Another limitation of the current review is the use of studies concerning experimentally induced acute pancreatitis because this disease does not necessarily mimic spontaneous disease. In particular, the severity of the induced disease, which is typically described as severe acute pancreatitis with tissue necrosis and mortality rates up to 86% [61], is not directly comparable with the veterinary clinical setting, where the severity of acute pancreatitis varies from subclinical to very severe.

Two meta-analyses were found during the literature search, one on humans and one on rodents; however, they were excluded and only relevant individual studies were retained. The human meta-analysis included 5 studies, but only one was included in this review [38] because the others were only published in Chinese and hence excluded based on the English language criterion [67]. Consistent with the current review, the human meta-analysis concluded that corticosteroid therapy may improve outcomes in patients with severe acute pancreatitis. From the meta-analysis on rodents, 6 studies were included [36, 41, 47, 48, 61, 62], the text for one study could not be retrieved, and the remaining studies included pretreatment with steroids and were thus excluded [68]. This meta-analysis also concluded that corticosteroids have beneficial effects on rodent animal models of severe acute pancreatitis.

None of the included studies presented sample size calculations; thus, the possibility of studies being underpowered cannot be ignored. Drop-outs were often poorly described or not described in the included studies. However, drop-outs were considered to have a minor influence on the results, especially for the experimental studies, where animals typically were closely followed for a short period of time (hours or a few days). Similarities at baseline were reasonable, and only 7 of the 31 studies rated poor or fair. Older human and canine studies presented less than adequate descriptions. The level of evidence was also reasonable among the studies, with just 1 study graded LOE IV.

Acute pancreatitis is known to be a very painful condition. As most of the experimental studies had been evaluated by an ethical committee, one might wonder why only 2 of the experimental studies used analgesics in addition to corticosteroids, which meant that all animals in the control groups were not provided pain relief. Pain could very possibly influence the disease outcome and results.

In this review, 5 disease outcome parameters were evaluated. Clinical scores were reported in 4 studies; however, they were all differently defined, thereby limiting any conclusions on this parameter.

CRP is a major acute phase protein in several species, including mice, humans and dogs. It is one of the most sensitive markers of inflammation in dogs and used to confirm the presence of underlying inflammation and monitor the response to therapy [69, 70]. Furthermore, CRP testing is widely used in clinical practice. Therefore, CRP was included as an outcome parameter. In earlier human guidelines on acute pancreatitis, a CRP level > 150 mg/L was indicative of severe pancreatitis [71]. However, later studies found that CRP measured at the first visit is not a reliable prognostic marker for human acute pancreatitis because CRP peak levels are reached only after 48–72 h [72]. Kuzi et al. [4] found that CRP correlated significantly with cPLI and the canine acute pancreatitis severity score (CAPS), and CRP levels in survivors and nonsurvivors were similar on day 0 and day 1. Nevertheless, CRP levels measured 48–72 h following initial presentation might correlate with acute pancreatitis outcome. Holm et al. found that CRP was significantly elevated in dogs with spontaneous acute pancreatitis compared with healthy controls, and it decreased significantly from day 1 and 3 to day 5 in association with clinical improvement [73]. Sato et al. [74] found significantly lower CRP levels on days 3 and 4 in survivors compared with nonsurvivors among dogs with acute pancreatitis. In the present review, one human study [37], one canine study [57] and one rodent study [44] followed CRP over time. In the human study, there was a significant difference in CRP levels between HC-treated and nontreated patients at 48 h [37], and Okanishi et al. [57] found a significant difference between prednisolone-treated and nontreated dogs with acute pancreatitis on day 3. However, whether these improvements in CRP levels reflect a true reduction in inflammation and disease severity or a direct suppression of IL-6, which is the main inducer of hepatic CRP production, is not known. Many other parameters have been reported to be improved by corticosteroid treatment and could have been evaluated, including ascites [50], acute respiratory distress syndrome [38, 40], time to vasopressor cessation [37], arachidonic acid breakdown products [61], amylase, lipase, TNFα, and IL-6 levels [46, 51], and morphological changes of the lungs [54].

The reason why corticosteroids may positively affect the development of acute pancreatitis could be related to findings in human studies of relative adrenal insufficiency (RAI), which is also called critical illness-related corticosteroid insufficiency (CIRCI). Clinical studies have found that human patients suffering from severe acute pancreatitis have low corticosteroid baseline levels and/or poor response to ACTH stimulation. RAI/CIRCI in severe acute pancreatitis patients has been associated with higher clinical severity scores, pancreatic necrosis, need for surgical intervention and higher mortality [75, 76]. These findings, along with the abovementioned human meta-analysis by Dong et al. [67], have led to the cautious recommendation of corticosteroid therapy for severe acute pancreatitis in humans by some authors [77]. However, to the authors knowledge, this recommendation has not yet been implemented in any official treatment guidelines of human acute pancreatitis. Additionally, whether RAI is present in dogs with acute pancreatitis and whether it relates to survival is still controversial [78, 79].

As mentioned in the introduction, it is believed that CAP becomes life-threatening with the development of disseminated intravascular coagulation and multiple organ failure [2], and it could therefore be speculated that the use of corticosteroids in the treatment of CAP could be of particular benefit in severe cases at risk of developing these complications. Recent evidence has shown that corticosteroid treatment is beneficial for COVID-19 patients needing oxygen therapy or ventilation but not for patients managing without such therapy [80]. A common feature of human patients with severe acute pancreatitis and COVID-19 is the excessive formation of neutrophil extracellular traps (NETs) by activated neutrophils, leading to thrombosis and multiorgan damage [81, 82]. NETosis has been recognised in several species, including humans, mice and dogs [83]. Human patients with acute pancreatitis have increased levels of circulating NETs compared with healthy controls [84], and in mice, NETs can directly induce trypsin formation; moreover, mitigation of NET formation reduces pancreatic inflammation and lung damage [85]. Corticosteroids have been shown to downregulate NETs in the lungs of an experimental equine model of asthma [86], and in humans with chronic inflammatory lung disease, NETs were decreased in patients who frequently used corticosteroid inhalers [87]. The important role of neutrophils in disease progression is further supported by the recent approval in Japan of the drug fuzapladib sodium hydrate (BRENDA™Z, Ishihara Sangyo Kaisha Ltd. Osaka, Japan) for improving clinical signs in the acute phase of pancreatitis in dogs [88]. Fuzapladip sodium hydrate prevents the translocation of neutrophils from blood vessels to the extravascular tissue. It is not as broad-acting as corticosteroids and may have fewer side effects; however, until it is approved for global use, early short-term corticosteroid treatment could be considered for mitigating NET formation.

In addition to the anti-inflammatory effect, corticosteroids may also act as procoagulants, which could be an unwarranted effect in patients with acute pancreatitis. Therefore, based on current data, specific CAP subgroups may respond differently to corticosteroid treatment. Whether subgroups exist and how they should possibly influence recommendations on the dosage, treatment initiation and duration needs further investigation. When considering corticosteroid dosage and treatment duration, practitioners should keep in mind that studies have found evidence of corticosteroids causing or increasing the risk of acute pancreatitis [21, 22].

## Conclusions

The current review of 31 studies evaluating corticosteroid treatment of acute pancreatitis provides fair evidence indicating the beneficial effect of corticosteroids on the duration of hospitalisation and circulating CRP levels. With respect to mortality rate and histopathological severity, a clear conclusion is more difficult to reach. Good evidence has been provided for a decreased mortality rate with corticosteroid treatment, while at the same time, good evidence has been provided of a lack of significant effect. Good evidence has been provided for a lower histopathological score of pancreatic changes with corticosteroid treatment, while fair evidence has also been provided for a lack of difference.

These findings lead to the cautious conclusion that additional treatment with corticosteroids in the acute/acute-on-chronic phase of canine pancreatitis might have a positive influence on disease outcome.

Because the evidence in this review was gathered for several species and included experimentally induced pancreatitis, specific treatment recommendations cannot be provided regarding the type, timing and dosage for dogs with CAP in the clinical setting.

In conclusion, although corticosteroid treatment of CAP may be beneficial, this review recommends larger randomised controlled studies in dogs with spontaneously occurring acute/acute-on-chronic pancreatitis to further elucidate the effect of corticosteroid treatment, determine the type, dose and length of corticosteroid treatment and identify possible CAP patient subgroups that could possibly benefit.

## Supplementary Information

**Additional file 1.** Search strategy. A list of search terms used in the database literature search is provided to elucidate the search process. The list is uploaded separately in Microsoft Word format.

## Data Availability

All data are included in the article. Information on excluded studies can be obtained from the corresponding author upon reasonable request.
