# Supplementary material for: Corticosteroid treatment for acute/acute-on-chronic experimental and naturally occurring pancreatitis in several species: a scoping review to inform possible use in dogs
Source: Acta Vet Scand. 2021 Jul 13;63:28. doi: 10.1186/s13028-021-00592-0 (PMC8276032; doi:10.1186/s13028-021-00592-0)
Supplement: Supplementary file 1 — Additional file 1. Search strategy. A list of search terms used in the database literature search is provided to elucidate the search process. The list is uploaded separately in Microsoft Word format. [file 13028_2021_592_MOESM1_ESM.docx]

Additional file 1. Search strategy used in the databases

Search strategy;

1. Steroid*
2. Corticosteroid*
3. Cortiso*
4. Glucocorticoid*
5. Dexamethason*
6. Hydrocortiso*
7. Predniso*
8. Methylpredniso*
9. 1 or 2 or 3 or 4 or 5 or 6 or 7 or 8
10. Acute pancreatiti*
11. Acute on chronic pancreatiti*
12. Acute-on-chronic pancreatiti*
13. 10 or 11 or 12
14. 9 and 13
15. Remove duplicates from 14
16. Limit 15 to English language
